# Évaluation des effets de la chirurgie sur la qualité de vie des patients présentant une hydrocèle opérée à l’hôpital général de Tiassalé (Côte d’Ivoire)

**DOI:** 10.48327/mtsi.v6i3.2026.776

**Published:** 2026-07-29

**Authors:** Christiane Antoinette DJOMAN, Brahima KOUMA, Karim DOUMBIA, Ama Marie Noëlle ANO, Kokora Franck EKOU, Orsot TETCHI, Yao Eugène KONAN, Daniel Ekra KOUADIO, Issaka TIEMBRE

**Affiliations:** 1 UFR Sciences médicales, Université Félix Houphouët Boigny de Cocody, Abidjan, Côte d’Ivoire; 2 Programme national de lutte contre les maladies tropicales négligées à chimiothérapie préventive, Abidjan, Côte d’Ivoire; 3 Hôpital général de Tiassalé, Côte d’Ivoire

**Keywords:** Hydrocèle, Chirurgie, Qualité de vie, Tiassalé, Côte d’Ivoire, Afrique subsaharienne, Hydrocele, Surgery, Quality of life, Tiassalé, Côte d’Ivoire, Sub-Saharan Africa

## Abstract

**Contexte:**

Une campagne de chirurgie gratuite a été menée pour améliorer les conditions de vie des patients atteints d’hydrocèle. Dans ce cadre, une étude a été réalisée afin d’évaluer les effets de cette chirurgie sur la qualité de vie des patients opérés à l’hôpital général de Tiassalé.

**Patients et méthode:**

Une étude rétrospective à deux mesures, avant et après la chirurgie, a été réalisée du 1er octobre 2024 au 30 juin 2025. Les paramètres étudiés étaient la reprise de l’activité professionnelle, de l’activité sexuelle, et la satisfaction du patient. Les données ont été collectées à 1, 6 et 12 mois après la chirurgie, à partir d’une fiche d’enquête après l’obtention du consentement éclairé des patients, puis saisies avec Excel 2013 et enfin analysées à l’aide du logiciel R version 4.3.2.

**Résultats:**

Sur 72 patients opérés, 66 ont été retenus. La moyenne d’âge était de 46 ans (30-59 ans). La majorité était constituée de planteurs (82 %) et vivait en couple (67 %). La durée moyenne d’évolution de l’hydrocèle était de 9,2 ans (IC 95 % : 7,9-12 ans). Avant la chirurgie, les patients avaient signalé différentes gênes : esthétique (85 %), au travail (74 %), et dans l’activité sexuelle (68 %). Les cures ont été réalisées sous anesthésie locale à la xylocaïne 2 % pour 79 % des patients. La résection vaginale a été réalisée avec succès pour 89 % d’entre eux. La quantité de liquide retirée a été supérieure ou égale à 250 cc chez 70 % des personnes. Après la correction chirurgicale, 11 % d’entre elles ont repris leur activité professionnelle à 1 mois puis 82 % à 6 mois et 98 % à 12 mois. La reprise de l’activité sexuelle a été obtenue chez 3 % des patients à 1 mois, 68 % à 6 mois et 89 % à 12 mois. Après la chirurgie, la proportion de patients qui a affirmé être très satisfaite était de 74 % à 1 mois, 92 % à 6 mois et 85 % à 12 mois.

**Conclusion:**

La chirurgie de l’hydrocèle a permis de restaurer la dignité des patients par la reprise et l’amélioration de leur vie sociale et sexuelle.

## Abstract (en)

**Background:**

A free surgery campaign was conducted to improve the quality of life for patients with hydrocele. As part of this effort, a study was conducted to evaluate the effects of the surgery on patients’ quality of life at Tiassalé General Hospital.

**Patients and methods:**

A retrospective longitudinal study with two measurement points, before and after surgery, was conducted from October 1, 2024, to June 30, 2025. The parameters studied were return to work, resumption of sexual activity, and patient satisfaction. Data were collected one, six, and twelve months after surgery using a questionnaire after informed consent was obtained from patients. The data were then entered into Excel 2013 and analyzed using R software version 4.3.2.

**Results:**

Of the 72 patients who underwent surgery, 66 were included in the study. The mean age was 46 years (range: 30–59 years). Most were farmers (82%) who lived with a partner (67%). The mean duration of the hydrocele was 9.2 years (95% CI: 7.9–12 years). Before surgery, patients reported various discomforts, including cosmetic issues (85%), discomfort at work (74%), and discomfort during sexual activity (68%). The procedures were performed under local anesthesia with 2% Xylocaine in 79% of patients. Vaginal resection was successfully performed in 89% of patients. The volume of fluid removed was 250 cc or more in 70% of patients. Following surgical correction, 11% of patients returned to work at one month, 82% at six months, and 98% at 12 months. Resumption of sexual activity was achieved by 3% of patients at one month, 68% at six months, and 89% at 12 months. The proportion of patients who reported being very satisfied was 74% at one month, 92% at six months, and 85% at 12 months.

**Conclusion:**

Hydrocele surgery restored patients’ dignity by enabling them to resume and improve their social and sexual lives.

**Figure:**
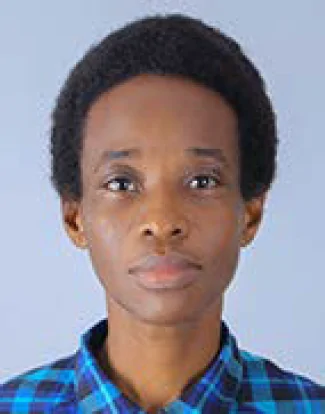

## Introduction

Le lymphœdème est une augmentation durable du volume d’une partie du corps, le plus souvent la jambe ou le bras, liée à une accumulation de lymphe. L’hydrocèle est une accumulation de liquide séreux dans la tunique vaginale entourant le testicule, provoquant un gonflement du scrotum [7]. Dans les régions où l’hydrocèle est endémique, ces deux affections sont le plus souvent des manifestations cliniques de la phase chronique de la filariose lymphatique (FL). Elles entraînent non seulement des handicaps physiques, mais également psychologiques et sociaux, qui impactent négativement la qualité de vie des personnes atteintes et de leur entourage [7]. Les hommes atteints d’hydrocèle endurent une défiguration, une perte d’estime de soi, une altération de la fonction sexuelle et une perte de statut social. En plus de ces conséquences physiques et sociales, elles perpétuent la pauvreté en réduisant la productivité et en augmentant les dépenses de santé des personnes touchées [7]. Sur le plan épidémiologique, *Wuchereria bancrofti* est souvent responsable de la maladie en Afrique. Ce parasite est transmis à l’homme par la piqûre de moustiques, principalement *Culex quinquefasciatus* [6,7].

Entre 2007 et 2020, sur 34 pays de la région Africaine où la FL est endémique, 26 ont notifié avoir enregistré 148 114 cas de lymphœdèmes et 137 696 cas d’hydrocèles, et seulement 4 ont signalé avoir offert des soins aux patients en 2020 [8]. En Côte d’Ivoire, 99 des 113 districts sanitaires que compte le pays sont endémiques à la FL dont 90 coendémiques avec l’onchocercose. Tous les districts sanitaires du pays nécessitant l’administration de masse de médicaments ont bénéficié en 2020 de traitements et tous ont obtenu une couverture thérapeutique efficace égale ou supérieure à 65 % [8].

Parmi ces districts sanitaires, figure celui de Tiassalé. La prévalence de base de la FL était de 32,7 %. Après cinq programmes d’administration de masse de médicaments (AMM), l’enquête d’évaluation de la transmission (TAS1) puis l’enquête de surveillance (TAS2) ont noté chacune une prévalence de moins de 1 %, témoignant ainsi de l’interruption de la transmission de la maladie dans ce district.

Pour parvenir à l’élimination de cette maladie, l’OMS recommande, en plus de l’AMM, la réduction des souffrances et des incapacités liées à la maladie par l’hygiène et les soins de la peau chez les patients atteints de lymphœdème, ainsi que par des interventions chirurgicales pour les hommes souffrant d’hydrocèle [9]. Ces deux recommandations sont appuyées par la sensibilisation des communautés sur la maladie.

Bien que la chirurgie soit reconnue comme essentielle, peu d’études en Côte d’Ivoire ont mesuré ses effets sur la qualité de vie des patients opérés. L’objectif général de cette étude était d’évaluer les effets de la chirurgie des hydrocèles vaginales sur la qualité de vie des patients opérés dans le district sanitaire de Tiassalé entre 2022 et 2023.

## Méthode

Il s’agissait d’une étude longitudinale portant sur des données d’une campagne de chirurgie de l’hydrocèle vaginale de l’adulte organisée du 7 décembre 2022 au 15 janvier 2023. Elle a été réalisée dans le service de chirurgie de l’hôpital général de Tiassalé, sous la coordination du district sanitaire. D’une superficie de 4350 km2, le district sanitaire héberge une population estimée en 2024 à 386966 habitants (Institut national de la statistique).

La population étudiée était constituée de personnes de sexe masculin, âgées de 15 ans et plus, présentant une hydrocèle vaginale probablement d’origine filarienne, associée ou non à une comorbidité (hernie inguino-scrotale). Ces personnes avaient consulté le service de chirurgie de l’hôpital général de Tiassalé, site choisi pour l’organisation de la campagne de chirurgie gratuite de l’hydrocèle.

Tous les patients présentant une grosse bourse sans rapport avec une hydrocèle de la vaginale (hernie inguino-scrotale, kyste du cordon spermatique, cancer du testicule…), et tout patient opéré n’ayant pas fait la totalité des trois consultations de suivi post-opératoire n’ont pas été inclus dans cette étude. Les efforts de recherche par appels téléphoniques des numéros donnés par les patients euxmêmes et leurs parents proches lors de leur inclusion dans le programme opératoire, et les visites à domicile faites par les agents de santé communautaire n’ont pas permis de retrouver les personnes absentes aux consultations de suivi. L’échantillon de départ était constitué de 72 patients répondant aux critères de sélection. Six patients n’ayant pas pu réaliser la totalité des consultations de suivi post-opératoire à 1, 6 et 12 mois (Fig. 1), l’échantillon de l’étude a porté sur 66 patients.

**Figure 1:**
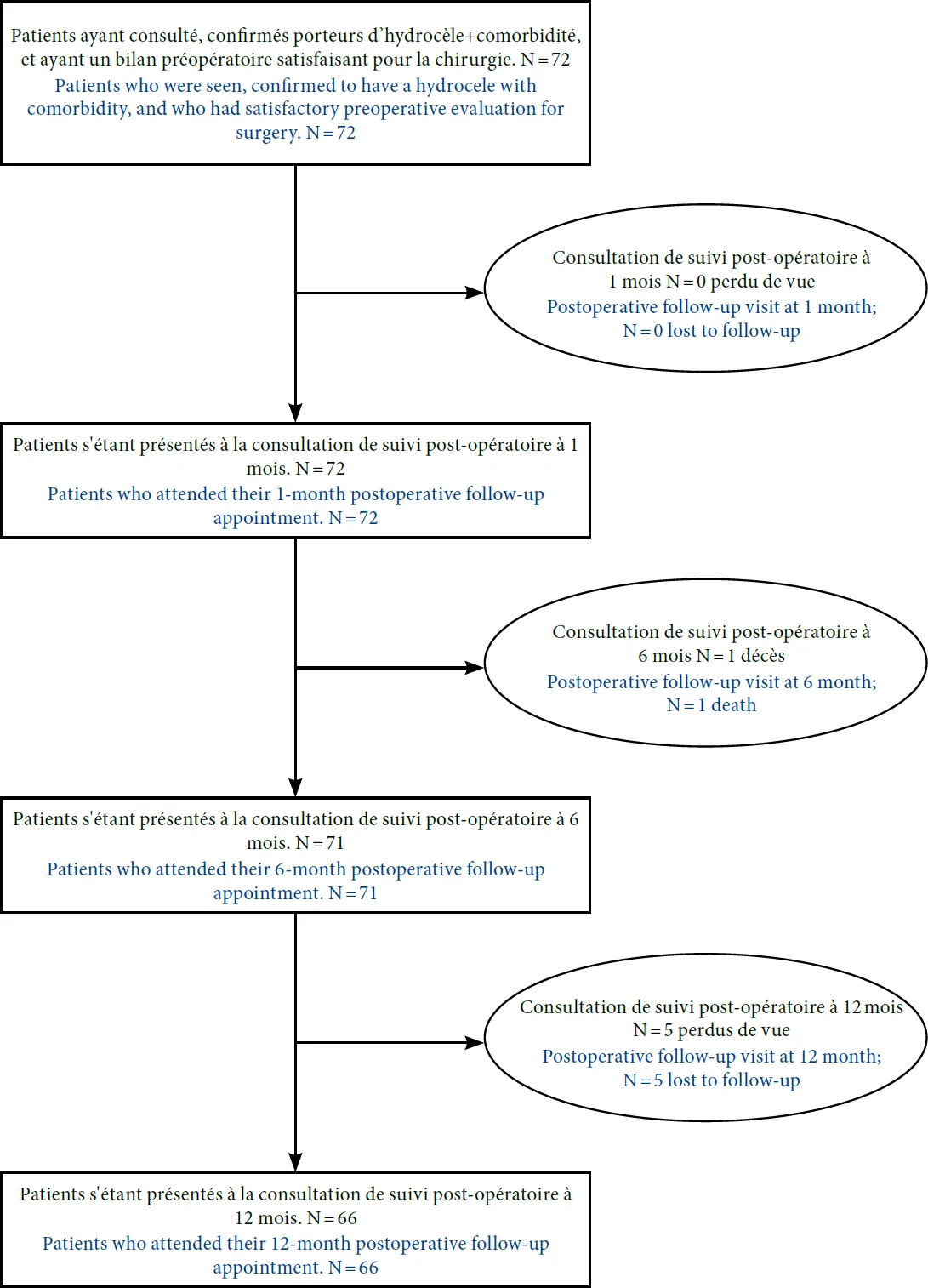
Organigramme d’inclusion et d’exclusion des sujets de l’étude

Les données ont été collectées au cours de la consultation clinique de confirmation du chirurgien, du bilan pré-opératoire échographique et biologique, des consultations de suivi post-opératoires à 1, 6 et 12 mois, ainsi qu’à partir de la fiche clinique du patient, du registre d’anesthésie et du registre de chirurgie. L’évaluation du niveau de gêne ressenti a été faite selon les critères suivants : important, peu important et pas du tout. Les paramètres de qualité de vie considérés ont été : i) la reprise de l’activité professionnelle, ii) la reprise de l’activité sexuelle, iii) l’amélioration de la vie sexuelle, et iv) la satisfaction du patient. Le niveau de satisfaction des patients après la chirurgie a été ainsi apprécié : très satisfait, peu satisfait et insatisfait. Les patients ont été interrogés directement et leurs réponses ont été consignées. Les données ont été saisies avec Excel 2013 et analysées avec le logiciel R version 4.3.2. Les variables quantitatives ont été décrites sous formes de moyennes et d’écart type. Les variables qualitatives ont été décrites sous formes de proportions.

Le protocole d’étude a été soumis au Comité national d’éthique des sciences de la vie et de la santé pour accord pour sa mise en œuvre (sous le no 126-22/MSHPCMU/CNESVS-kp en date du 31 août 2022).

Chaque patient a donné son consentement éclairé pour être opéré (signature ou empreinte digitale sur la fiche). La confidentialité et l’anonymat ont été préservés dans l’exploitation des documents qui ont servi à ces travaux.

## Résultats

Sur 72 patients opérés, 66 ont été retenus au terme de 12 mois de suivi après la chirurgie, soit 91 %. Leur âge moyen était de 45,6 ans ± 11,2 ans avec des extrêmes de 21 et 74 ans. Les planteurs (82 %) étaient les plus nombreux (n=54) et la plupart (n = 44) était en couple (67 %). La tranche d’âge la plus représentée (n = 27) était celle de 30 à 44 ans (41 %) suivie de celle de 45 à 59 ans (n = 25) soit 39 %.

La durée moyenne d’évolution de l’hydrocèle était de 9,2 ans (IC 95 % : 7,9-12 ans). L’hydrocèle gauche était prédominante chez 24 enquêtés (36 %), suivie de la forme bilatérale pour 23 d’entre eux (35 %). En outre, 7 patients (11 %) présentaient aussi une hernie inguino-scrotale. Les cures ont été réalisées sous anesthésie locale à la xylocaïne 2 % chez 79 % des patients (n = 52). La résection vaginale a été réalisée avec succès chez 89 % d’entre eux (n = 59). La quantité de liquide était supérieure ou égale à 250 cc pour 70 % des patients (n = 46). En ce qui concerne les gestes associés, 1 cas d’orchidectomie unilatérale a été réalisé pour fonte testiculaire purulente (2 %). Les 7 cas de hernie inguino-scrotale ont bénéficié d’une cure herniaire associée à leur hydrocélectomie (11 %).

Avant la chirurgie, la santé physique et mentale des patients était affectée par une gêne esthétique, une gêne de travail et une gêne de l’activité sexuelle (Fig. 2).

**Figure 2:**
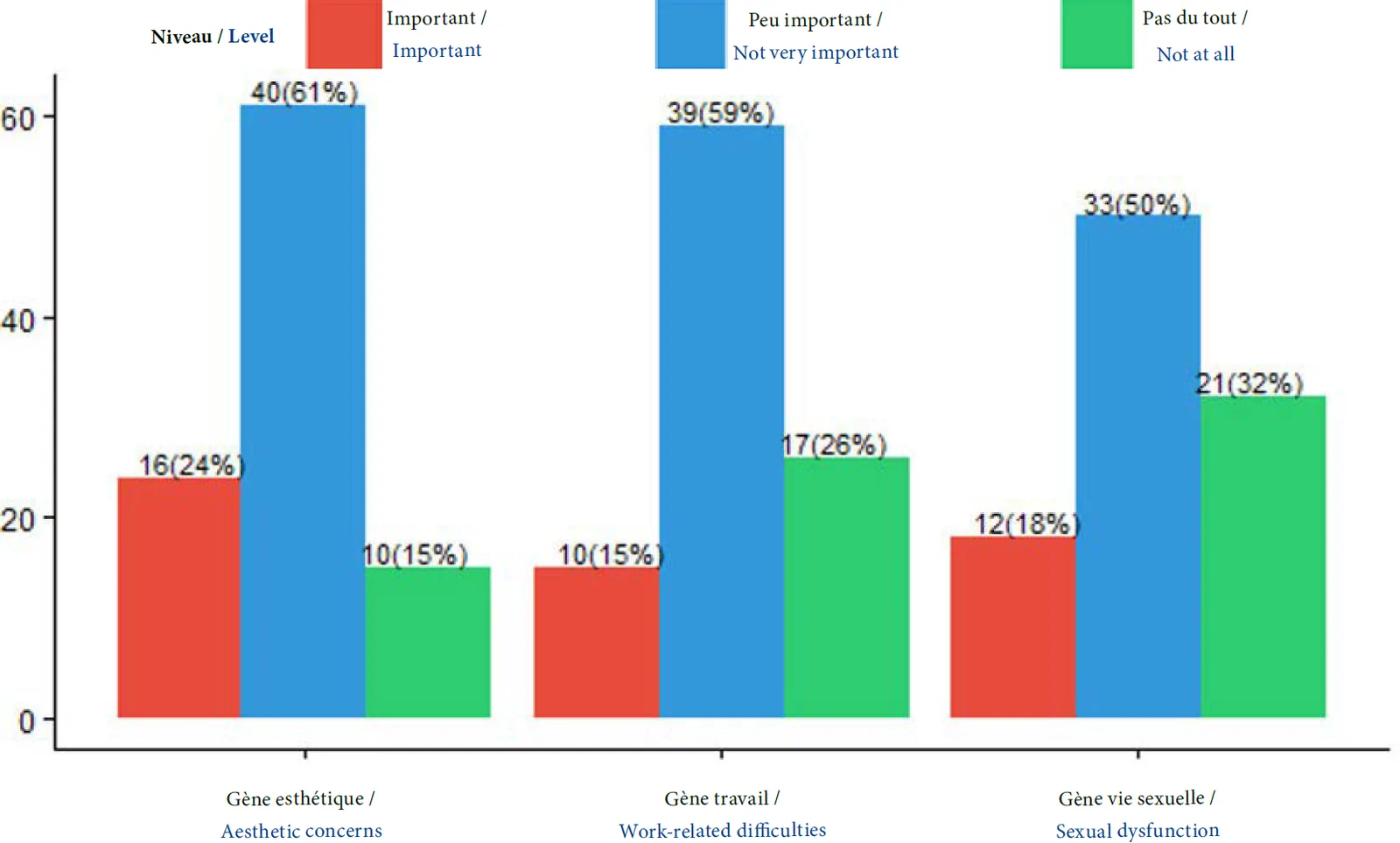
Répartition des patients opérés d’hydrocèle à l’hôpital général de Tiassalé selon la gêne esthétique, la gêne du travail et la gêne de la vie sexuelle, avant la chirurgie (2022-2023)

Un mois après la chirurgie, 89 % des patients n’ont présenté aucune complication (n = 59). Cependant, 9 % d’entre eux ont eu une surinfection (n = 6) et un patient a présenté un hématome enkysté. Six mois après la chirurgie, aucun n’a eu de complication. Le délai de reprise des activités est précisé dans la Figure 3 et la Figure 4.

**Figure 3:**
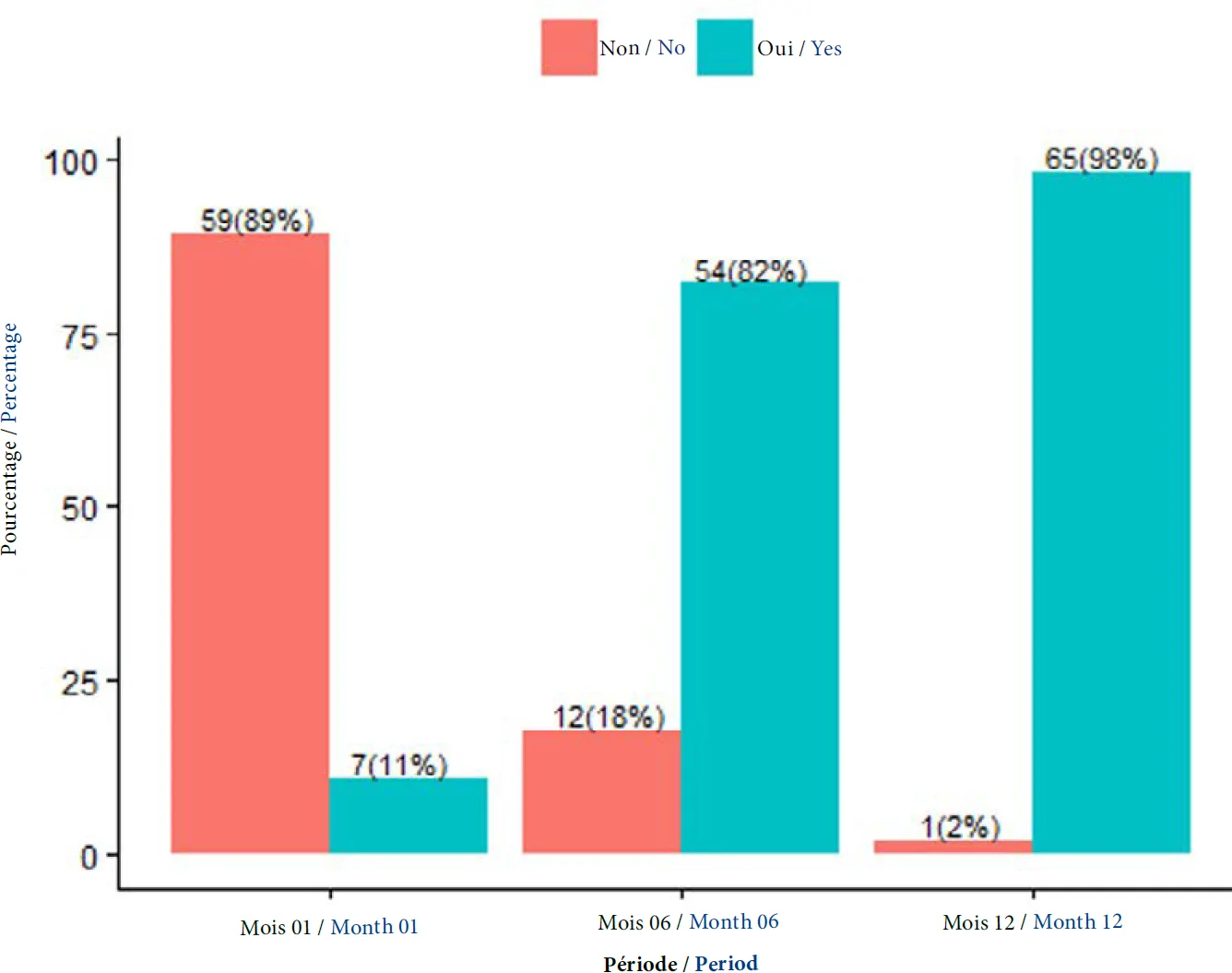
Répartition des patients opérés d’hydrocèle à l’hôpital général de Tiassalé selon la reprise de l’activité professionnelle après la chirurgie (2022-2023)

**Figure 4:**
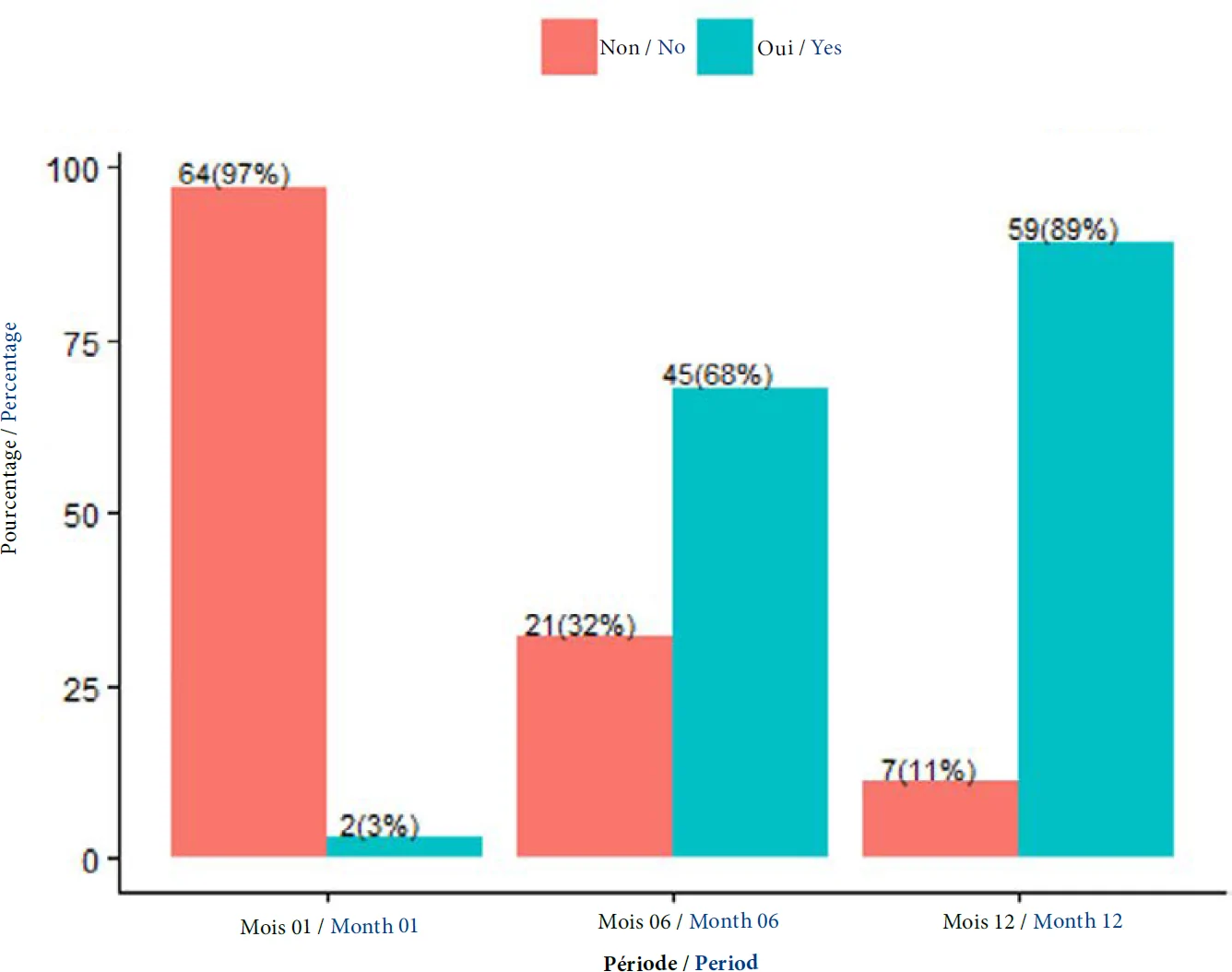
Répartition des patients opérés d’hydrocèle à l’hôpital général de Tiassalé selon la reprise de l’activité sexuelle après la chirurgie (2022-2023)

Le niveau de satisfaction des patients après la chirurgie est présenté en Figure 5.

**Figure 5:**
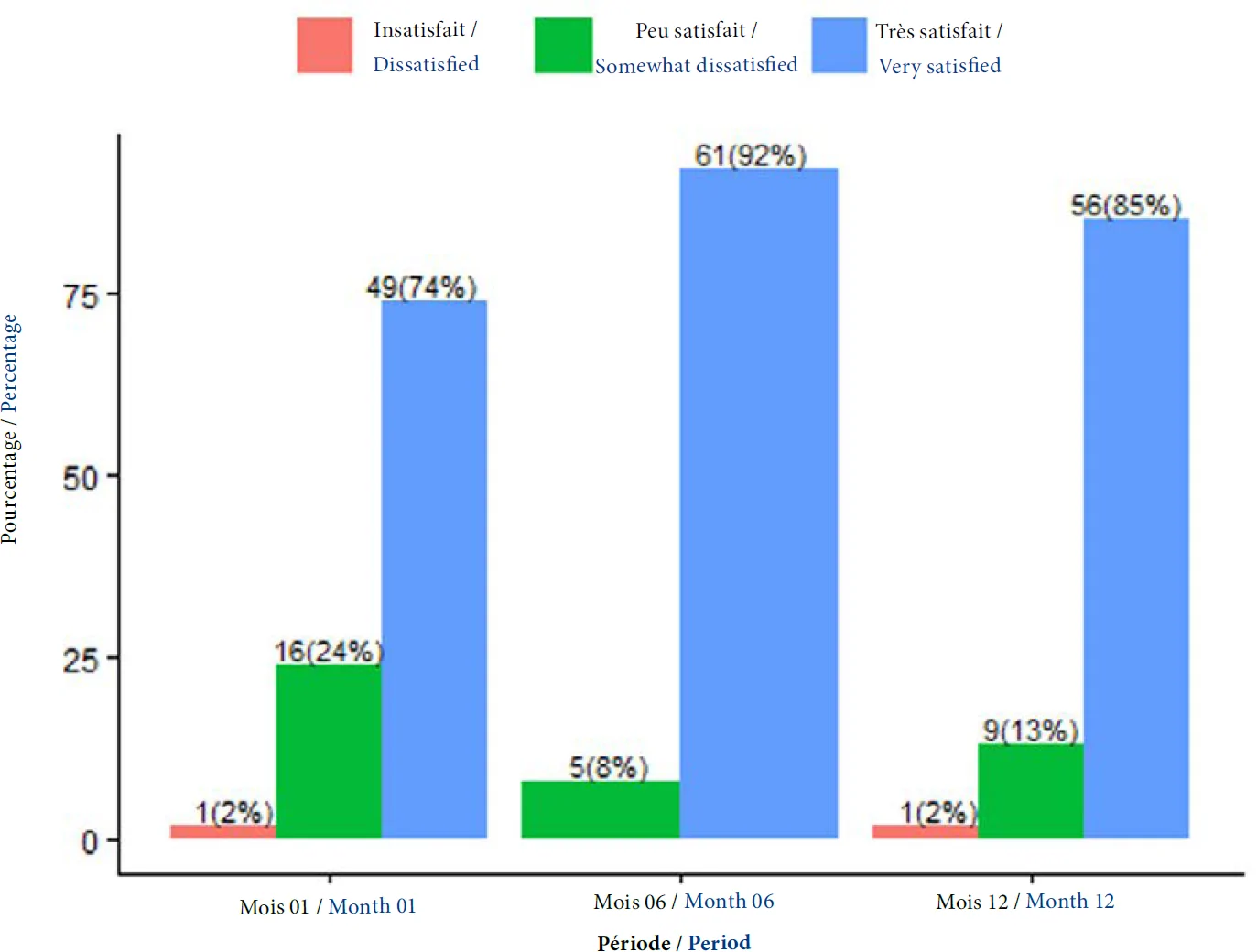
Répartition des patients opérés d’hydrocèle à l’hôpital général de Tiassalé selon leur état de satisfaction après la chirurgie (2022-2023)

## Discussion

Cette étude a permis d’évaluer les bénéfices de la chirurgie de l’hydrocèle vaginale sur le bienêtre des patients dans un district sanitaire de Côte d’Ivoire. Sa contextualisation s’avère nécessaire pour comprendre le cadre dans lequel elle s’est inscrite.

En tenant compte des réalités sanitaires dans lesquelles elle a été menée, cette étude présente des limites. Le diagnostic de la FL par les tests immunologiques (*filariasis test strip*), la recherche de microfilaire *W. bancrofti* dans un prélèvement sanguin nocturne (22 heures à 2 heures du matin) et dans le liquide d’hydrocèle n’ont pu être réalisés. Cependant, au regard de la forte endémicité de la FL dans ce district sanitaire, une hydrocèle observée chez tous les patients âgés de 15 ans et plus a été considérée comme d’origine filarienne due à *W. bancrofti*. La classification de Capuano évaluant la gravité de l’hydrocèle n’a pu être réalisée avant l’intervention chirurgicale. Enfin, l’analyse de la qualité de vie des patients opérés s’est limitée aux déclarations verbales renseignées sur la fiche clinique, ce qui pourrait avoir un impact sur l’appréciation globale de l’effet de la chirurgie. Malgré ces insuffisances de données, l’intérêt de cette étude demeure important.

Le recours tardif aux soins chez ces patients s’expliquerait par plusieurs raisons dont la crainte de la stigmatisation sociale et le manque de res-sources financières. Selon plusieurs auteurs, la honte et les tabous liés aux « us et coutumes», la crainte de la perte de la fonction érectile et de la stérilité à la suite du traitement chirurgical ainsi que le manque de moyens financiers sont autant d’éléments qui amèneraient les patients à retarder la prise en charge [3,12].

Avant la chirurgie, la proportion de patients qui avaient signalé une importante gêne esthétique était élevée. Cette gêne, en rapport avec le volume de l’hydrocèle (quantité de liquide supérieure ou égale à 250 cc chez plusieurs des patients), pouvait entraîner une modification des habitudes vestimentaires obligeant ces patients à porter un boubou ou nouer un pagne. Elle est à l’origine de moqueries et source d’isolement comme mentionnées dans des études au Ghana qui ont affirmé que: « L’hydrocèle en raison de son volume limite parfois les mouvements et est parfois source de moqueries» [1,4]. Cette situation peut également affecter l’état de santé mentale des patients avec pour conséquence une baisse de l’estime de soi pouvant évoluer jusqu’au suicide [2].

Au niveau social, l’hydrocèle constituait une gêne importante au travail pour les patients qui pour la plupart étaient jeunes et exerçaient le métier de planteur. Ceci a pu entraîner une perte de productivité et par conséquent, un manque de ressources financières. Cet impact sur le travail en rapport avec le volume et la pesanteur de l’hydrocèle a été noté dans des travaux au Ghana, en Côte d’Ivoire, au Burkina Faso et au Malawi [1,5,10,11]. L’étude réalisée au Malawi, avait rapporté que : « La perte annuelle moyenne de revenus attribuée à l’hydrocèle est de 126 $ US et la valeur actualisée moyenne de la perte de revenus à vie pour ces hommes de moins de 65 ans est de 1 684 $ US » [11].

La gêne sexuelle signalée par 68 % des patients, résulterait également du volume et du poids de l’hydrocèle. Une étude réalisée en Inde avait mis en évidence que 94 % des patients avaient affirmé connaître des difficultés lors des rapports sexuels et que 94 % des épouses de patients atteints d’hydrocèles avaient déclaré être insatisfaites lors de ces rapports [2]. Cet impact de l’hydrocèle sur la vie sexuelle des patients a été également confirmé par des études au Ghana et au Burkina Faso, qui avaient mentionné que les manifestations chroniques de la FL pouvaient entraîner des difficultés psycho-sociales telles que la réduction des perspectives de mariage, la dysfonction sexuelle et le divorce [1,10].

L’intervention chirurgicale s’est traduite par une amélioration de la qualité de vie des patients. Celleci découle, d’abord, de la correction chirurgicale réalisée qui a atténué le poids du liquide d’hydrocèle, et a permis à 98 % des patients de reprendre leurs activités professionnelles 12 mois après l’intervention. Cette amélioration des activités professionnelles après chirurgie a été également décrite par des auteurs au Ghana qui ont noté des améliorations remarquables de la capacité de travail des patients après la chirurgie. Ils ont souligné que « Les patients satisfaits ont déclaré avoir eu une restauration de l’estime de soi, leur permettant ainsi de participer plus activement aux activités communautaires » [1].

De même, la chirurgie a permis à 89 % des patients de reprendre 12 mois après une activité sexuelle. Une étude antérieure menée en Côte d’Ivoire avait trouvé une amélioration de la vie sexuelle chez 93,8 % des patients opérés [5]. Elle avait rapporté que : « Les patients ont dit avoir une amélioration de leurs érections, des rapports sexuels plus agréables et une perte de honte lors des rapports sexuels». Concernant la satisfaction, les patients ont affirmé dans leur majorité, avoir été très satisfaits à 1, 6 et 12 mois après la chirurgie. Le faible nombre de récidives et de complications post-opératoires contribue également à améliorer leur qualité de vie.

## Conclusion

L’hydrocèle vaginale est reconnue comme pouvant être une complication importante de l’infestation de l’homme par *W. bancrofti*. Pathologie fréquente dans les pays tropicaux et subtropicaux, elle impacte négativement la vie sociale, sexuelle et économique du patient et de la communauté dans laquelle il vit. En raison de ces répercussions sur la santé, l’OMS l’a incluse au nombre des maladies tropicales négligées posant un problème de santé publique. Cette campagne de chirurgie d’hydrocèle a permis de restaurer la dignité des patients par la reprise et l’amélioration de leur vie sociale et sexuelle, de faire naître en eux une estime de soi, de les rendre plus productifs, et de les sortir eux et leurs communautés de l’extrême pauvreté.

## Sources de financement

La chirurgie des hydrocèles et le suivi post-opératoire à un mois ont été financés par *SIGHTSAVERS* à travers le programme national de lutte contre les maladies tropicales négligées à chimiothérapie préventive (PNLMTN-CP) de Côte d’Ivoire. Le suivi post-opératoire à 6 et 12 mois a été assuré par le ministère en charge de la santé de Côte d’Ivoire.

## Contribution des auteurs et autrices

Kouma Brahima et Doumbia Karim ont recruté et opéré les patients d’hydrocèle et réalisé la collecte de données; Djoman Christiane Antoinette et Kouma Brahima ont élaboré le protocole de recherche et rédigé le manuscrit; Ano Ama Marie Noëlle, Ekou Franck, Tetchi Orsot, Konan Eugène, Ekra Kouadio Daniel et Tiembré Issaka ont révisé le manuscrit. Tous les auteurs ont déclaré également avoir lu et approuvé la version finale du document.

## Déclaration de liens d’intérêt

Aucun lien d’intérêt n’a été déclaré.
