# Supplementary material for: Assessment of the effects of hydrocele surgery on the quality of life of patients at Tiassalé General Hospital (Côte d’Ivoire)
Source: Med Trop Sante Int. 2026 Jul 29;6(3):mtsi.v6i3.2026.776. [Article in French] doi: 10.48327/mtsi.v6i3.2026.776 (PMC13697897; doi:10.48327/mtsi.v6i3.2026.776)
Supplement: Related texts

# Research article: Assessment of the effects of hydrocele surgery on the quality of life of patients at Tiassalé General Hospital (Côte d’Ivoire)

## Introduction

Lymphedema is a persistent increase in the volume of a body part, most commonly the leg or arm, caused by a buildup of lymph. A hydrocele is an accumulation of serous fluid in the tunica vaginalis that surrounds the testicle, causing the scrotum to swell [7]. In regions where hydroceles are endemic, these two conditions are often clinical manifestations of the chronic phase of lymphatic filariasis (LF). These conditions result in physical, psychological, and social disabilities that negatively impact the quality of life of those affected and their families [7]. Men with hydroceles suffer from disfigurement, loss of self-esteem, impaired sexual function, and loss of social status. These conditions perpetuate poverty by reducing productivity and increasing healthcare costs [7]. From an epidemiological perspective, *Wuchereria bancrofti* is often responsible for the disease in Africa. This parasite is transmitted to humans through mosquito bites, primarily from *Culex quinquefasciatus* [6,7].

From 2007 to 2020, 26 of the 34 countries in the African region where LF is endemic reported 148,114 cases of lymphedema and 137,696 cases of hydrocele. However, only four of these countries reported providing care to patients in 2020 [8]. In Côte d’Ivoire, 99 of the country’s 113 health districts are endemic for LF; 90 of these districts are also endemic for onchocerciasis. In 2020, all health districts requiring mass drug administration received treatment and achieved effective treatment coverage of 65% or higher [8].

Tiassalé is one of these health districts. The baseline prevalence of LF was 32.7%. Following five mass drug administration (MDA) programs, the transmission assessment surveys (TAS1 and TAS2) recorded prevalence rates of less than 1%, confirming the interruption of disease transmission in the district.

To eliminate this disease, the WHO recommends reducing the suffering and disability associated with it through hygiene and skin care for patients with lymphedema, as well as surgical interventions for men with hydrocele [9]. Community awareness campaigns about the disease support these two recommendations.

Although surgery is recognized as essential, few studies in Côte d’Ivoire have measured its effects on patients’ quality of life.

This study aimed to evaluate the impact of a vaginal hydrocele surgery campaign conducted between end of 2022 and beginning of 2023 on the quality of life of patients in the Tiassalé health district.

## Method

This was a longitudinal study based on data from a surgical campaign for adult vaginal hydrocele conducted from December 7, 2022, to January 15, 2023. The study was carried out in the surgery department of Tiassalé General Hospital under the health district’s coordination. The health district covers an area of 4,350 km² and had an estimated population of 386,966 in 2024 (National Institute of Statistics).

The study population consisted of males aged 15 years and older who presented with a vaginal hydrocele, likely of filarial origin, with or without a comorbid condition (inguinoscrotal hernia). These individuals sought care at Tiassalé General Hospital’s surgical department, the site selected for the free hydrocele surgery campaign.

Patients with a large scrotal swelling unrelated to a hydrocele (e.g., inguinoscrotal hernias, spermatic cord cysts, or testicular cancer) and patients who underwent surgery but did not complete all three postoperative follow-up visits were excluded from this study. Some efforts to locate patients who missed follow-up visits, including telephone calls to numbers provided by patients and their close relatives at enrollment and home visits by community health workers, were unsuccessful. The initial sample consisted of 72 patients who met the selection criteria. Since six patients were unable to complete all postoperative follow-up visits at 1, 6, and 12 months (Fig. 1), the final sample size was 66 patients.

**Figure 1:** Flowchart of inclusion and exclusion criteria for study participants

Data were collected during the surgeon’s confirmatory clinical consultation, the preoperative ultrasound and laboratory workup, and the postoperative follow-up visits at one, six, and twelve months. Data were also collected from the patient’s medical record, the anesthesia registry, and the surgical registry. The level of discomfort experienced was assessed according to the following criteria: significant, minor, or none.

Quality-of-life parameters considered were: 1) return to work, 2) resumption of sexual activity, 3) improvement in sexual life, and 4) patient satisfaction. Patient satisfaction after surgery was assessed as follows: very satisfied, somewhat satisfied, or dissatisfied. Patients were interviewed directly, and their responses were recorded. The data were entered using Excel 2013 and analyzed using R software, version 4.3.2. Quantitative variables were described in terms of means and standard deviations. Qualitative variables were described as proportions.

The study protocol was submitted to the National Ethics Committee for Life Sciences and Health for approval prior to implementation (under No. 126-22/MSHPCMU/CNESVS-kp dated August 31, 2022).

Each patient provided informed consent to undergo surgery (signature or fingerprint on the consent form). Confidentiality and anonymity were maintained in the analysis of the documents used for this study.

## Results

Of the 72 patients who underwent surgery, 66 were included in the analysis following a 12-month postoperative follow-up period, representing 91%. Their mean age was 45.6 years ± 11.2 years, with ages ranging from 21 to 74 years. Farmers (82%) were the most common (n = 54), and most (n = 44) were in a relationship (67%). The most represented age group (n = 27) was 30 to 44 years (41%), followed by 45 to 59 years (n = 25), accounting for 39%.

The average duration of the hydrocele was 9.2 years (95% CI: 7.9–12 years). A left-sided hydrocele was predominant in 24 respondents (36%), followed by a bilateral form in 23 of them (35%). In addition, 7 patients (11%) also had an inguinal-scrotal hernia. Surgery was performed under local anesthesia with 2% xylocaine in 79% of patients (n = 52). Vaginal resection was successfully performed in 89% of them (n = 59). The fluid volume was greater than or equal to 250 cc in 70% of patients (n = 46). Regarding associated procedures, one case of unilateral orchiectomy was performed for purulent testicular necrosis (2%). The seven cases of inguinal-scrotal hernia underwent hernia repair in conjunction with their hydrocelectomy (11%).

Prior to surgery, the patients’ physical and mental health was affected by cosmetic concerns, work-related issues, and sexual dysfunction (Fig. 2).

**Figure 2:** Distribution of patients who underwent hydrocele surgery at Tiassalé General Hospital based on aesthetic concerns, work-related difficulties, and sexual dysfunction prior to surgery (2022–2023)

One month after surgery, 89% of patients (n = 59) experienced no complications. However, 9% developed a secondary infection (n = 6), and developed an encapsulated hematoma. Six months after surgery, none of the patients had experienced any complications. Figure 3 and Figure 4 show the time to resumption of activities.

**Figure 3:** Distribution patients who underwent hydrocele surgery at Tiassalé General Hospital by return to work after surgery (2022–2023)

**Figure 4:** Distribution of patients who underwent hydrocele surgery at Tiassalé General Hospital by time since resuming sexual activity after surgery (2022–2023)

Figure 5 presents the level of patient satisfaction after surgery.

**Figure 5:** Distribution of patients who underwent hydrocele surgery at Tiassalé General Hospital by level of satisfaction after surgery (2022–2023)

## Discussion

This study evaluated the benefits of vaginal hydrocele surgery on patients’ well-being in a health district of Côte d’Ivoire. Understanding the context in which the study was conducted is necessary to grasp the setting.

Due to the health realities in which the study was conducted, it has limitations. Diagnosis of LF using immunological tests (e.g., filariasis test strips) and detection of *W. bancrofti* microfilariae in nocturnal blood samples (collected between 10 p.m. and 2 a.m.) and hydrocele fluid could not be performed. However, due to the high prevalence of LF in this health district, hydroceles observed in patients aged 15 years and older were considered to be of filarial origin due to *W. bancrofti*.

The Capuano classification system for assessing hydrocele severity could not be applied prior to surgery. Finally, the analysis of quality of life among patients who underwent surgery was limited to verbal reports recorded on the clinical form, which could impact the overall assessment of the surgery’s effectiveness. Despite these limitations in the data, this study remains significant.

Several factors can contribute to the delayed seeking of care among these patients, including fear of social stigma and a lack of financial resources. According to several authors, shame and taboos related to “customs and traditions,” as well as fear of loss of erectile function and infertility after surgical treatment, are all factors that may lead patients to delay seeking care [3,12].

Prior to surgery, a significant proportion of patients reported cosmetic distress. This distress, related to the size of the hydrocele (with fluid volume greater than or equal to 250 cc in many patients), can result in changes in clothing habits. These patients may be forced to wear a boubou or tie a loincloth. This can lead to ridicule and isolation, as noted in studies from Ghana: “Due to its size, a hydrocele sometimes restricts movement and is sometimes a source of ridicule” [1,4]. This situation can affect patients’ mental health, resulting in reduced self-esteem, which can progress to suicide [2].

On a social level, a hydrocele can significantly hinder patients at work, especially those who are young farmers. This may lead to a loss of productivity and consequently, a lack of financial resources. Studies conducted in Ghana, Côte d’Ivoire, Burkina Faso, and Malawi have noted this impact on work related to the size and weight of the hydrocele [1,5,10,11]. A study conducted in Malawi reported that: “The average annual loss of income attributed to hydrocele is US$126, and the average present value of lifetime income loss for men under 65 is US$1,684” [11].

The sexual discomfort reported by 68% of patients is thought to result from the size and weight of the hydrocele. A study conducted in India found that 94% of patients and 94% of their wives reported experiencing difficulties during intercourse [2]. Studies in Ghana and Burkina Faso also confirmed the impact of hydrocele on patients’ sex lives, noting that chronic manifestations of LF can lead to psychosocial difficulties, such as reduced marriage prospects, sexual dysfunction, and divorce [1,10].

Surgical intervention improved patients’ quality of life. This improvement stems from the surgical correction that alleviated the burden of hydrocele fluid, enabling 98% of patients to resume professional activities 12 months after the procedure. Authors in Ghana also described this improvement in professional activities following surgery, noting remarkable improvements in patients’ work capacity after surgery. The authors emphasized that satisfied patients reported a restoration of self-esteem, enabling them to participate more actively in community activities [1].

Similarly, the surgery enabled 89% of patients to resume sexual activity 12 months after the procedure. A previous study conducted in Côte d’Ivoire found an improvement in sexual life among 93.8% of patients who underwent surgery [5]. The study reported that: “Patients reported improved erections, more pleasurable sexual intercourse, and a reduction in feelings of shame during intercourse.” Regarding satisfaction, the majority of patients reported being very satisfied at one, six, and twelve months after surgery. The low number of recurrences and postoperative complications also contributed to an improved quality of life.

## Conclusion

Vaginal hydrocele is a well-recognized complication of *W. bancrofti* infection in men. It is a common condition in tropical and subtropical countries that negatively impacts the social, sexual, and economic lives of patients and their communities. Due to these health implications, the WHO has classified it as a neglected tropical disease that poses a public health problem. The hydrocele surgery campaign has helped patients regain their dignity, resume normal social and sexual lives, build self-esteem, become more productive, and lift themselves and their communities out of extreme poverty.

## Funding

Hydrocele surgery and one-month postoperative follow-up were funded by Sightsavers through the National Program for the Control of Neglected Tropical Diseases through Preventive Chemotherapy (PNLMTN-CP) in Côte d’Ivoire. Postoperative follow-up at six and twelve months was provided by the Ministry of Health of Côte d’Ivoire.

## Authors’ contributions

Kouma Brahima and Doumbia Karim recruited patients, performed surgeries, and collected data. Djoman Christiane Antoinette and Kouma Brahima developed the research protocol and drafted the manuscript. Ano Ama Marie Noëlle, Ekou Franck, Tetchi Orsot, Konan Eugène, Ekra Kouadio Daniel, and Tiembré Issaka reviewed the manuscript. All authors declared that they had read and approved the final version of the document.

## Conflicts of interest

The authors declare no conflicts of interest.
